# Supplementary material for: A bioinspired scaffold for rapid oxygenation of cell encapsulation systems
Source: Nat Commun. 2021 Oct 6;12:5846. doi: 10.1038/s41467-021-26126-w (PMC8494927; doi:10.1038/s41467-021-26126-w)
Supplement: Supplementary file 1 — Supplementary Information [file 41467_2021_26126_MOESM1_ESM.pdf]

Supplementary Information

A bioinspired scaffold for rapid oxygenation of cell encapsulation systems

Long-Hai Wang[1], Alexander Ulrich Ernst[1], Duo An[1], Ashim Kumar Datta[1], Boris Epel[2], Mrignayani Kotecha[3], and Minglin Ma[1]*

[1]Biological and Environmental Engineering, Cornell University, Ithaca, NY 14853, USA
[2]Department of Radiation and Cellular Oncology, The University of Chicago, Chicago, IL, 60637, USA
[3]O2M Technologies, LLC, Chicago, IL, 60612, USA

*Corresponding author. E-mail: mm826@cornell.edu

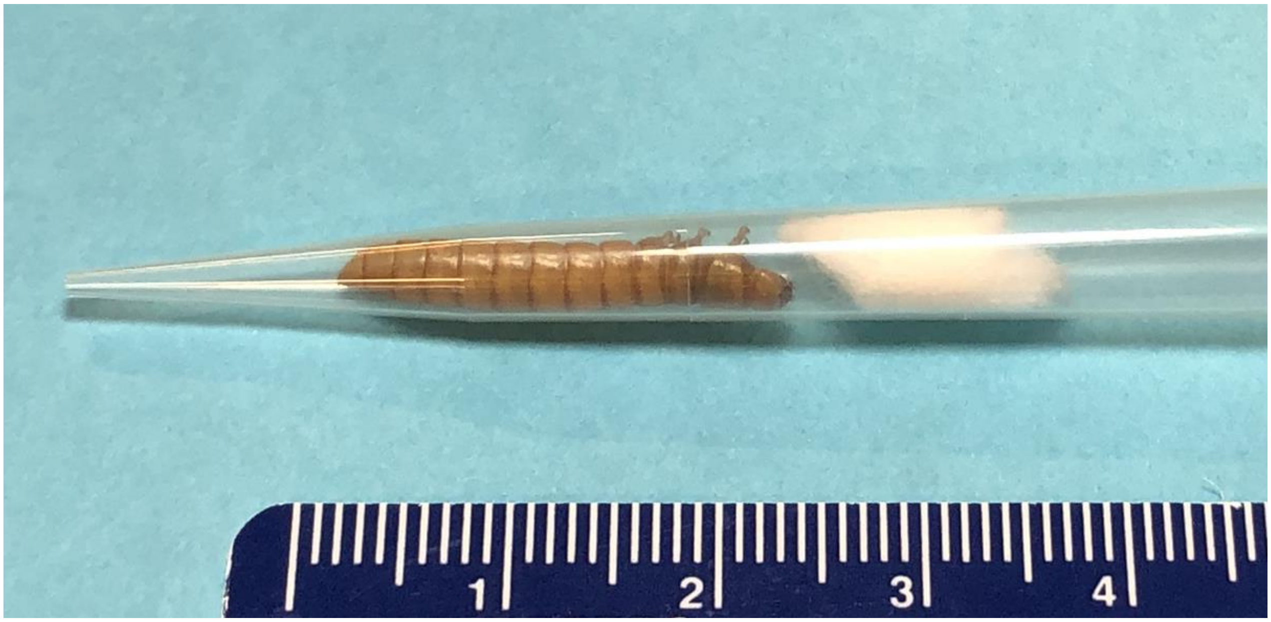

Supplementary Fig. 1 | A digital image of the mealworm in a pipet tip for Nano-CT scanning.

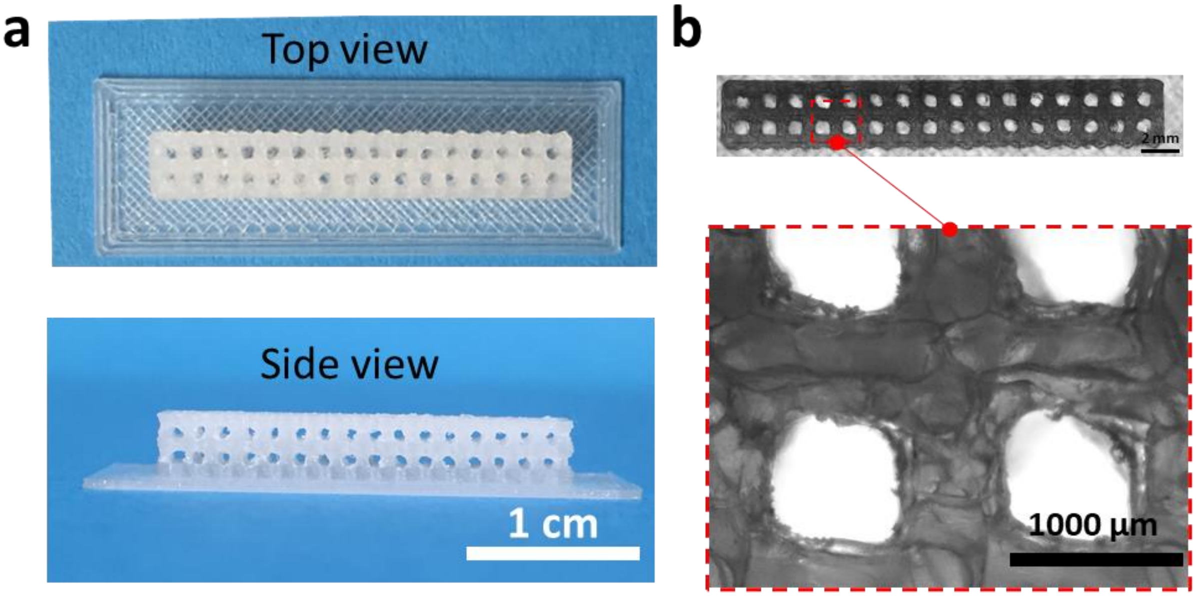

Supplementary Fig. 2 | a, **b** Digital images (**a**) and microscope images (**b**) of the 3D-printed PLA mold for fabrication of the two-layer ladder-like SONIC scaffold. One representative of 11 replicates is shown.

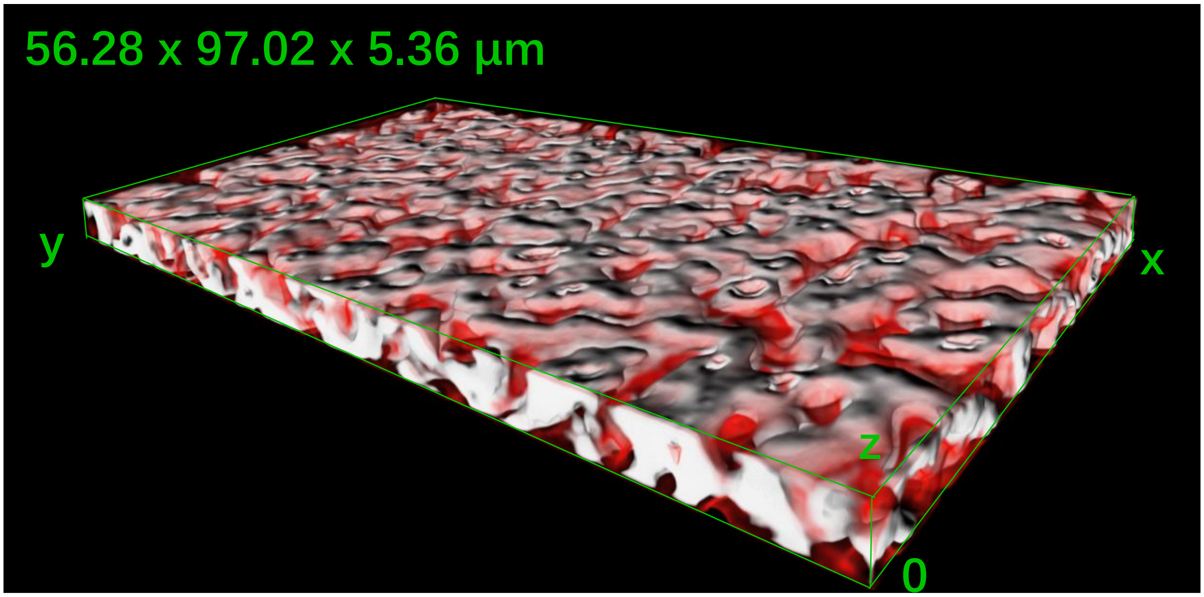

Supplementary Fig. 3 | 3D reconstruction of Nano-CT images of a selected region (56.28 × 97.02 × 5.36 µm) inside the SONIC scaffold showing the bicontinuous microstructure (the diffuse red coloring indicates the air phase in the porous skeleton).

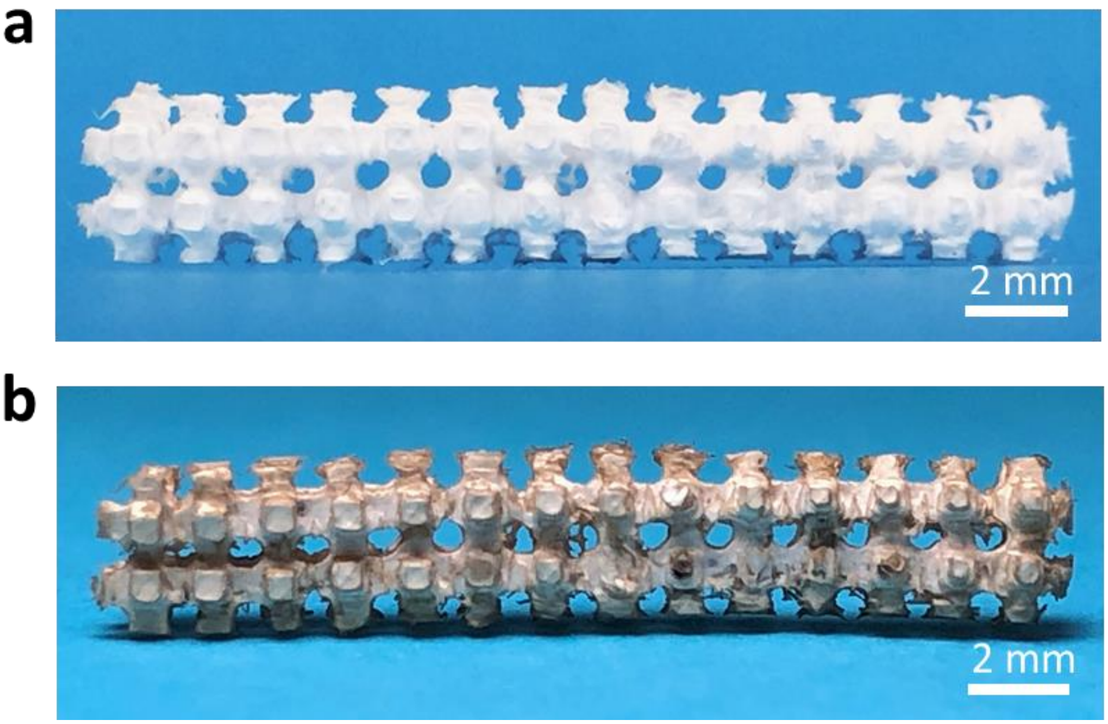

Supplementary Fig. 4 | a, **b** Digital images of the two-layer ladder-like SONIC scaffold before (**a**) and after (**b**) the surface hydrophilicity modification with polydopamine.

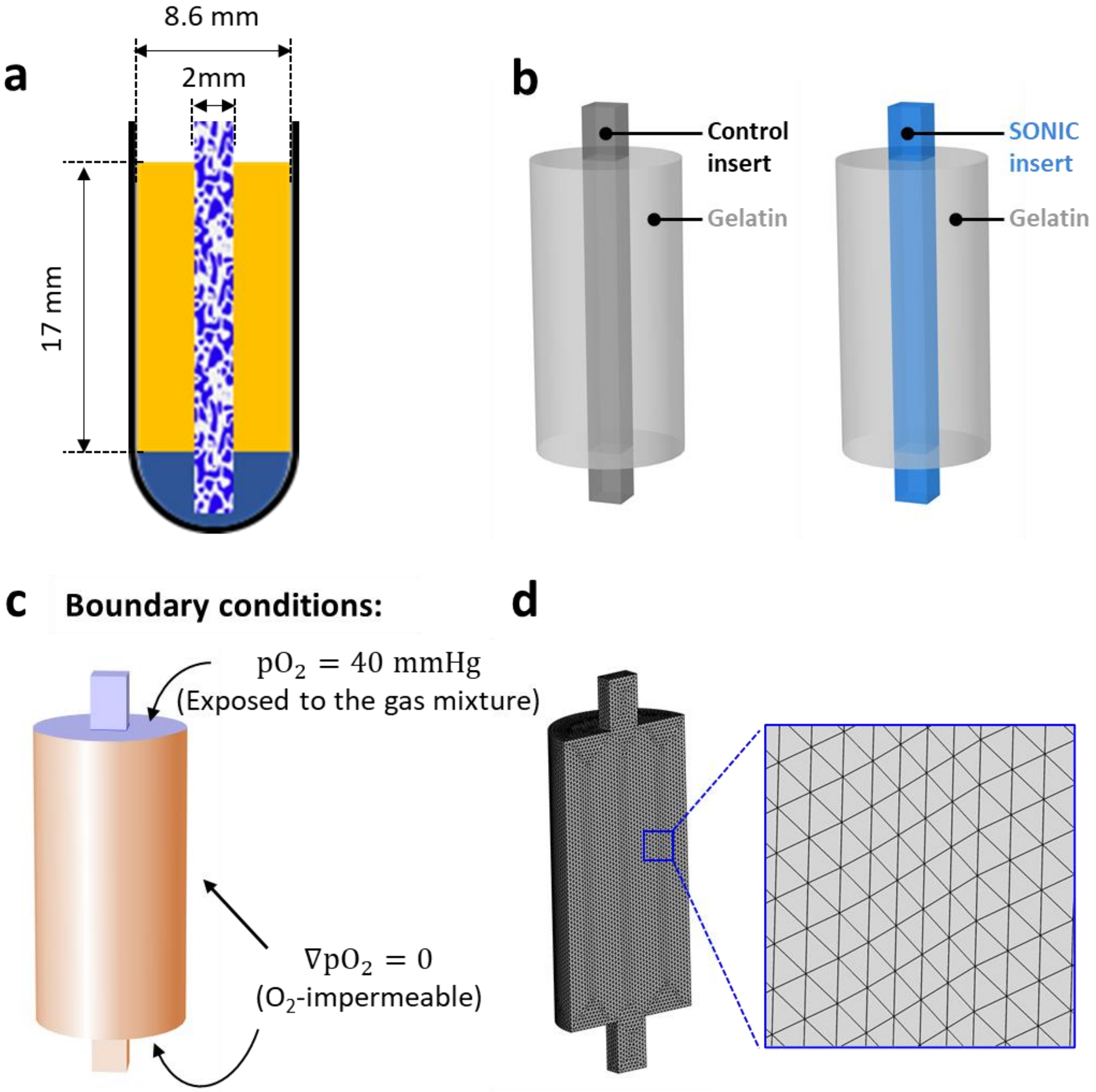

Supplementary Fig. 5 | a, **b** Dimensions (**a**) and schematic (**b**) representing the model geometry simulating the EPR diffusion tests. **c**, Boundary conditions: the top face of the gelatin and all exposed faces of the insert were set at a constant pO_2 of 40 mmHg while all remaining faces were set as a no-flux boundary. **d**, Images of the mesh implemented in the simulation.

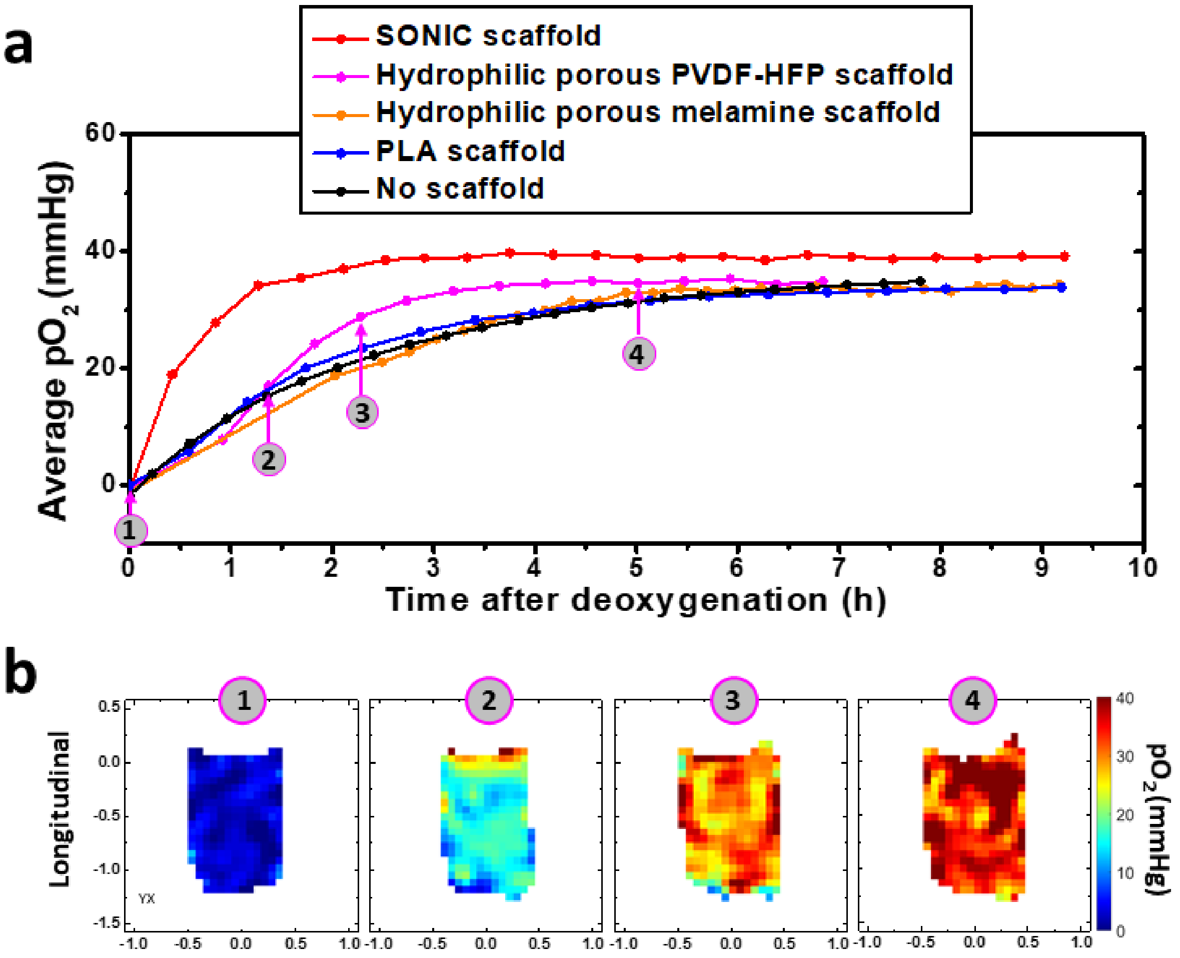

Supplementary Fig. 6 | a Average pO_2 in gelatin in the container over time with the SONIC scaffold, hydrophilic porous PVDF-HFP scaffold, hydrophilic porous melamine scaffold, PLA scaffold, and empty gelatin (no scaffold) after exposure to a gas mixture with a pO_2 of 40 mmHg. The SONIC scaffold and PLA scaffold data were reproduced from fig. 3d, f for comparison with additional controls. **b** pO_2 distributions on a tangential plane of control sample with the hydrophilic porous PVDF-HFP scaffold at different time points (indicated by the arrows in a), showing a slow equilibration via a top-to-bottom gradient. To prepare the hydrophilic porous PVDF-HFP control scaffold for the EPR test, ethanol was introduced into tris buffer (10 mM tris buffer, pH 8.5) with a volume ratio of 30% to get a dopamine (2 mg/mL) solution for the incubation of a PVDF-HFP scaffold (2 × 2 × 23 mm), which allows the polydopamine to coat the microporous channels and render them hydrophilic. Then, the polydopamine coated scaffold was treated with radio frequency plasma using argon and air gases to further ensure its hydrophilicity. The hydrophilic porous melamine scaffold was made from a melamine sponge material (www.spongeoutlet.com) using a 2 mm biopsy punch.

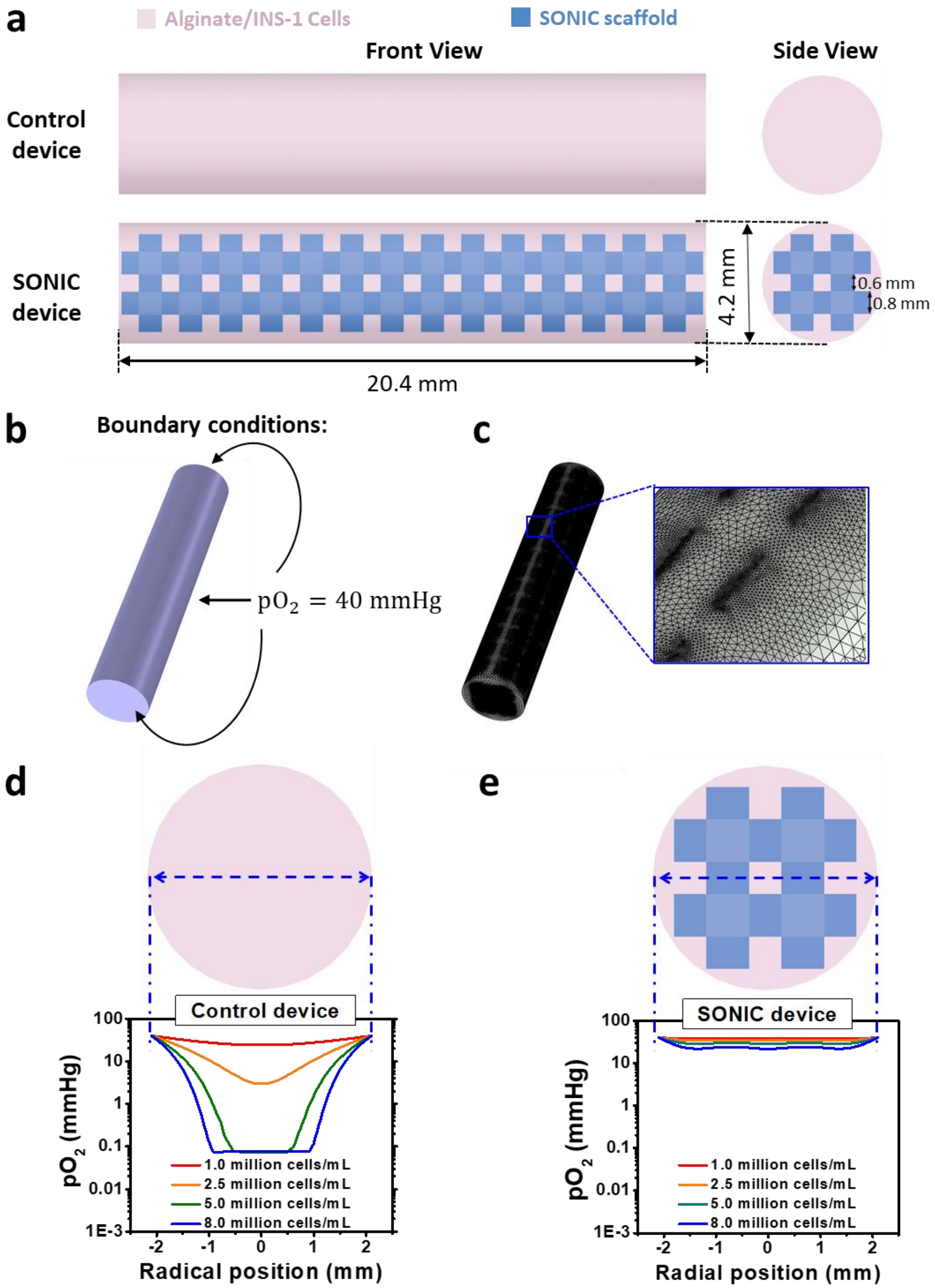

Supplementary Fig. 7 | a Schematic illustrating the geometry of the alginate/INS-1 encapsulation model, including the empty control (top), and SONIC device (bottom). **b** Boundary conditions: all faces were implemented as a constant pO_2 of 40 mmHg. **c** Images of the nonuniform mesh implemented in the simulation. **d**, **e** pO_2 along a radial line in the empty control device (**d**) and SONIC device (**e**) with multiple cell densities, showing large hypoxic regions in the center of the control device at high cell densities, while uniformly high pO_2 levels in the SONIC device.

Supplementary Table 1 | O_2 solubility, α, diffusivity, D, and permeability, (αD) in various potential scaffold materials.

Material	$\boldsymbol{\alpha}$ (mol/m³/Pa)	$\boldsymbol{D}$ (m²/s)	$(\boldsymbol{\alpha D})$ (mol/m/s/Pa)
**PLA**	4.50×10^{-5}	1.60×10^{-12}	7.20×10^{-17}
**Solid PVDF-HFP**	3.29×10^{-5}	3.50×10^{-12}	1.15×10^{-16}
**Hydrophilic porous PVDF-HFP/alginate†**	1.64×10^{-5}	1.89×10^{-9}	3.10×10^{-14}
**SONIC**	3.90×10^{-4}	1.80×10^{-5}	7.02×10^{-9}

†The coefficient for the hydrophilic porous PVDF-HFP/alginate was calculated by the composition volume fraction-weighted average of the coefficients for PVDF-HFP and alginate with the assumption that the PVDF-HFP's interior microporous channels were filled with alginate.

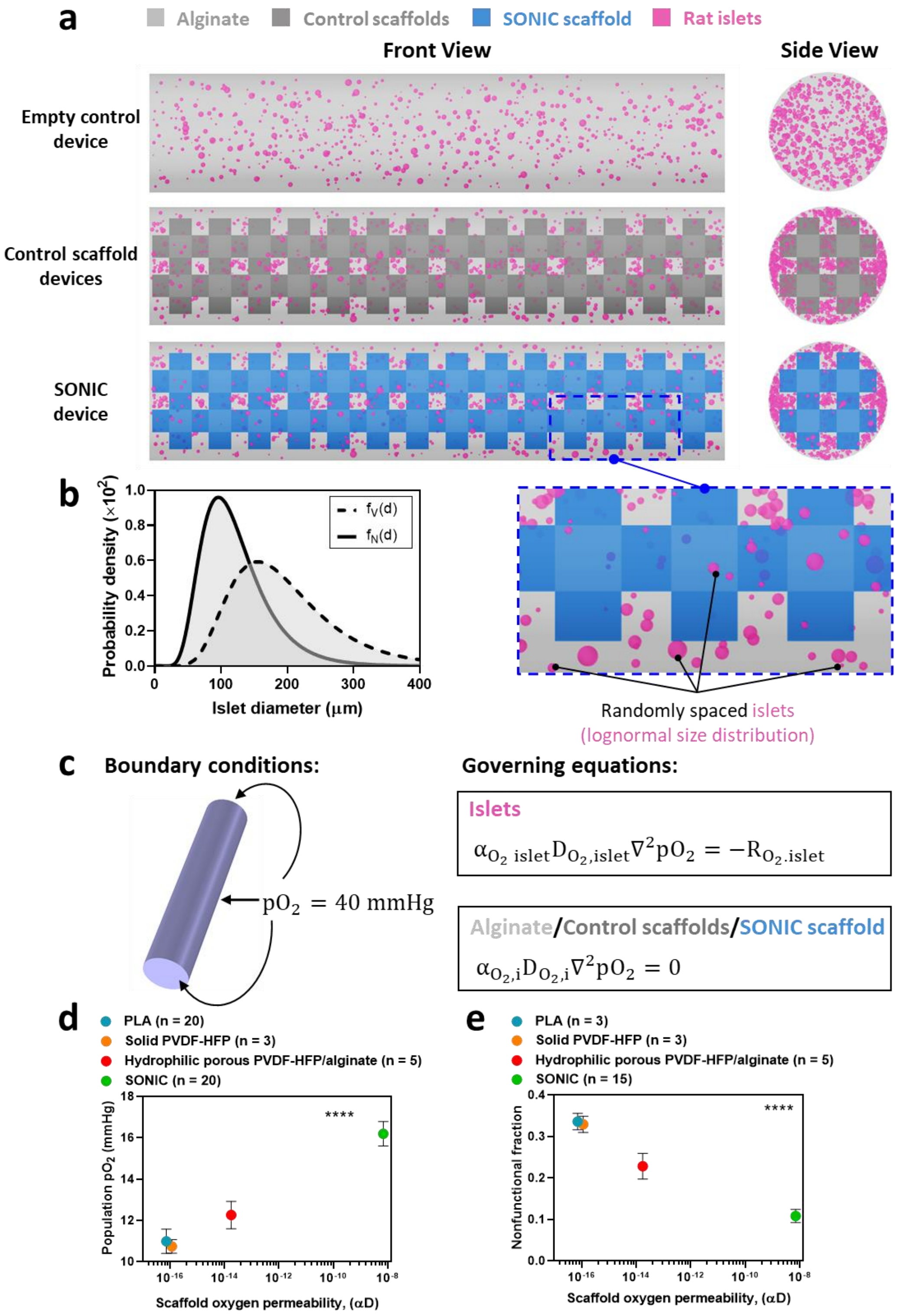

Supplementary Fig. 8 | a Schematic illustrating the geometry of the rat islet encapsulation model with size-distributed diameters (Fig. 4g–j, Supplementary Fig. 8), including the empty control device (top), control scaffold devices (middle; PLA scaffold, solid PVDF-HFP scaffold, or porous PVDF-HFP scaffold), and

SONIC device (bottom). **b** Probability density (left) of the size-distributed islets, showing the number-based probability density function (f_N) and volume-based probability density function (f_V) (see Equation 6 and related text); high magnification of a region of the modelled device (right), highlighting the size differences and random seeding of the simulated islets. **c** Boundary conditions: all faces were implemented as a constant pO_2 of 40 mmHg; Governing equations: O_2 transport in islets was governed by the diffusion-reaction mass balance equation whereas O_2 transport in all other domains was governed solely by diffusion (see the Computational Modeling section in the Methods for further details). **d**, **e** Dependence of islet oxygenation on scaffold O_2 permeability, (αD). Expected mean islet population pO_2 (**d**) and necrotic fraction (**e**) of 500 IEQ of rat islets in the device containing the SONIC scaffold or alternative control scaffolds including PLA, solid PVDF-HFP, or hydrophilic porous PVDF-HFP with its interior microporous channels filled with alginate, mean ± SD. Permeability values for each scaffold type are given in Table S1; d and e: ****$p < 0.0001$ (PLA, solid PVDF-HFP, and porous PVDF-HFP/alginate versus SONIC). Statistical tests in d and e were analyzed via a two-way ANOVA followed by Sidak's post hoc *p*-value adjustment for multiple comparisons.

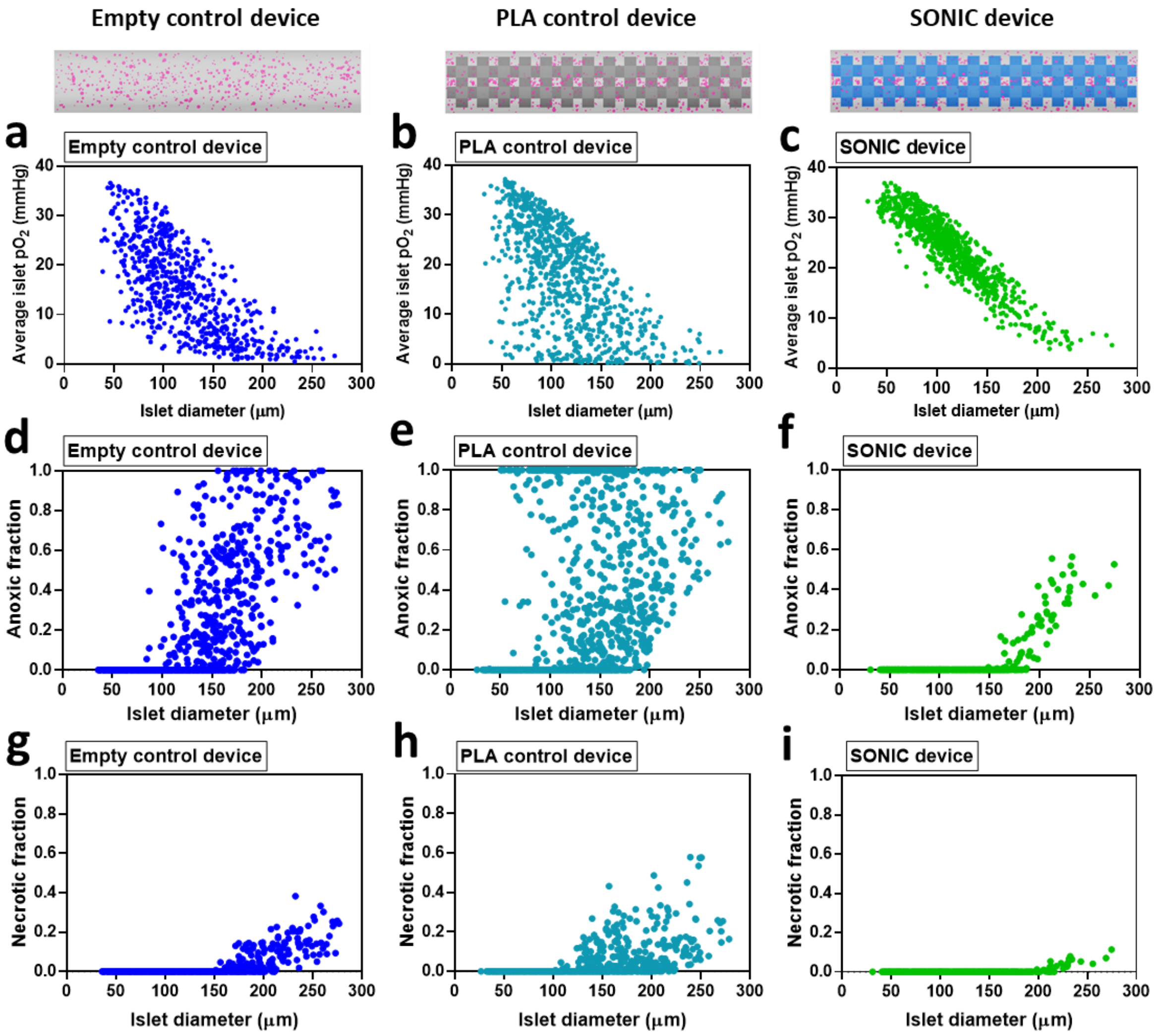

Supplementary Fig. 9 | a–c Scatter plots showing islet diameter versus average islet pO_2 of rat islets in empty control (**a**), PLA control (**b**), and SONIC device (**c**). **d–f** Scatter plots showing islet diameter versus anoxic (pO_2 < 4 mmHg) fraction of islets in empty control (**d**), PLA control (**e**), and SONIC device (**f**). **g–i** Scatter plots showing necrotic (pO_2 < 0.08 mmHg) volume fraction of islets versus islet diameter in empty control (**g**), PLA control (**h**), and SONIC device (**i**). Data was collected from one iteration of the simulation.

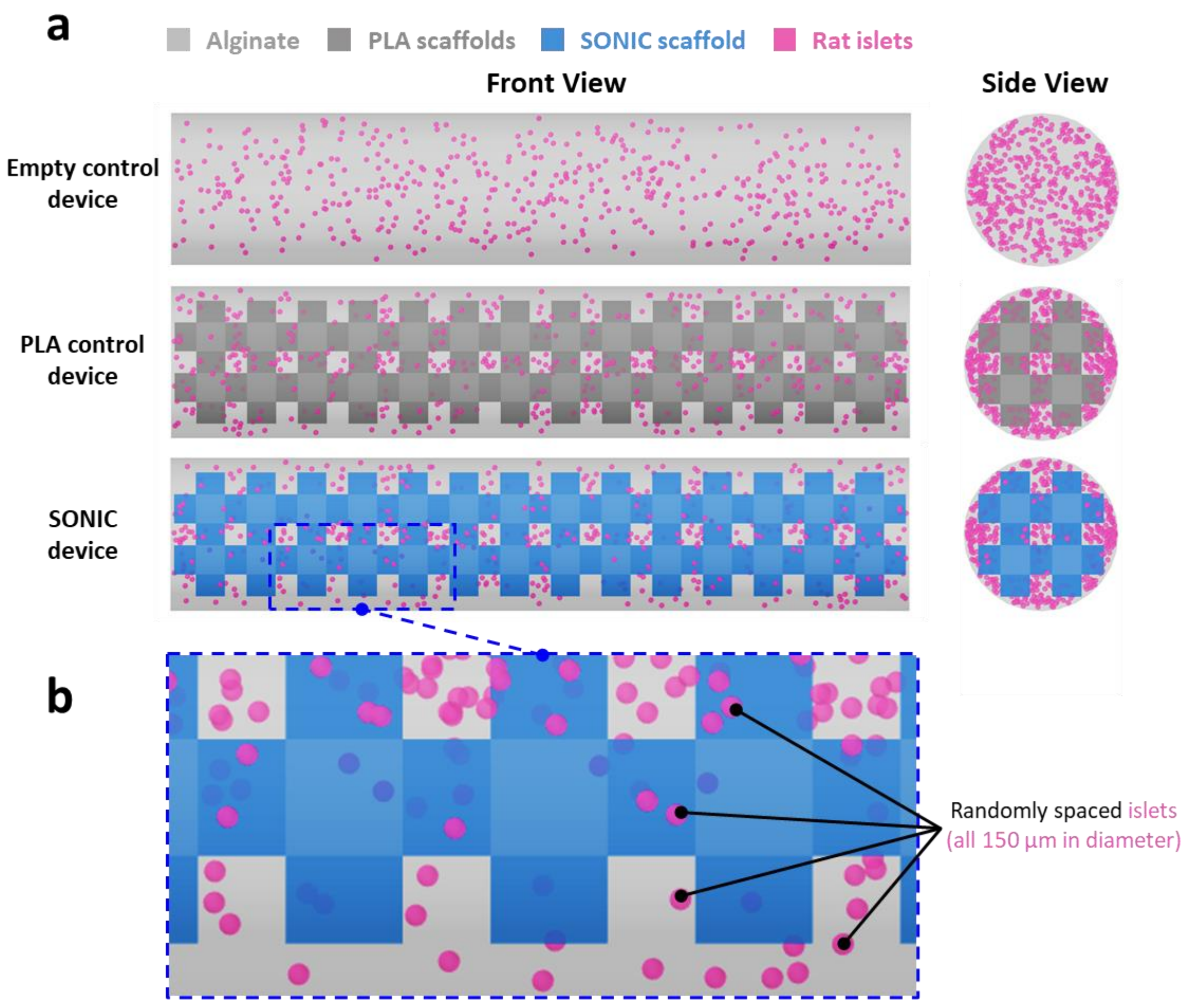

Supplementary Fig. 10 | a, **b** Schematic illustrating the geometry of the rat islet encapsulation model with uniformly distributed diameters (150 µm) (Fig. 4k, l), including the empty control (top), PLA control (middle), and SONIC device (bottom). Assuming all islets to be a uniform size isolated the effect of islet distance from the device exterior (*i.e.*, removed the size effect of islet oxygenation).

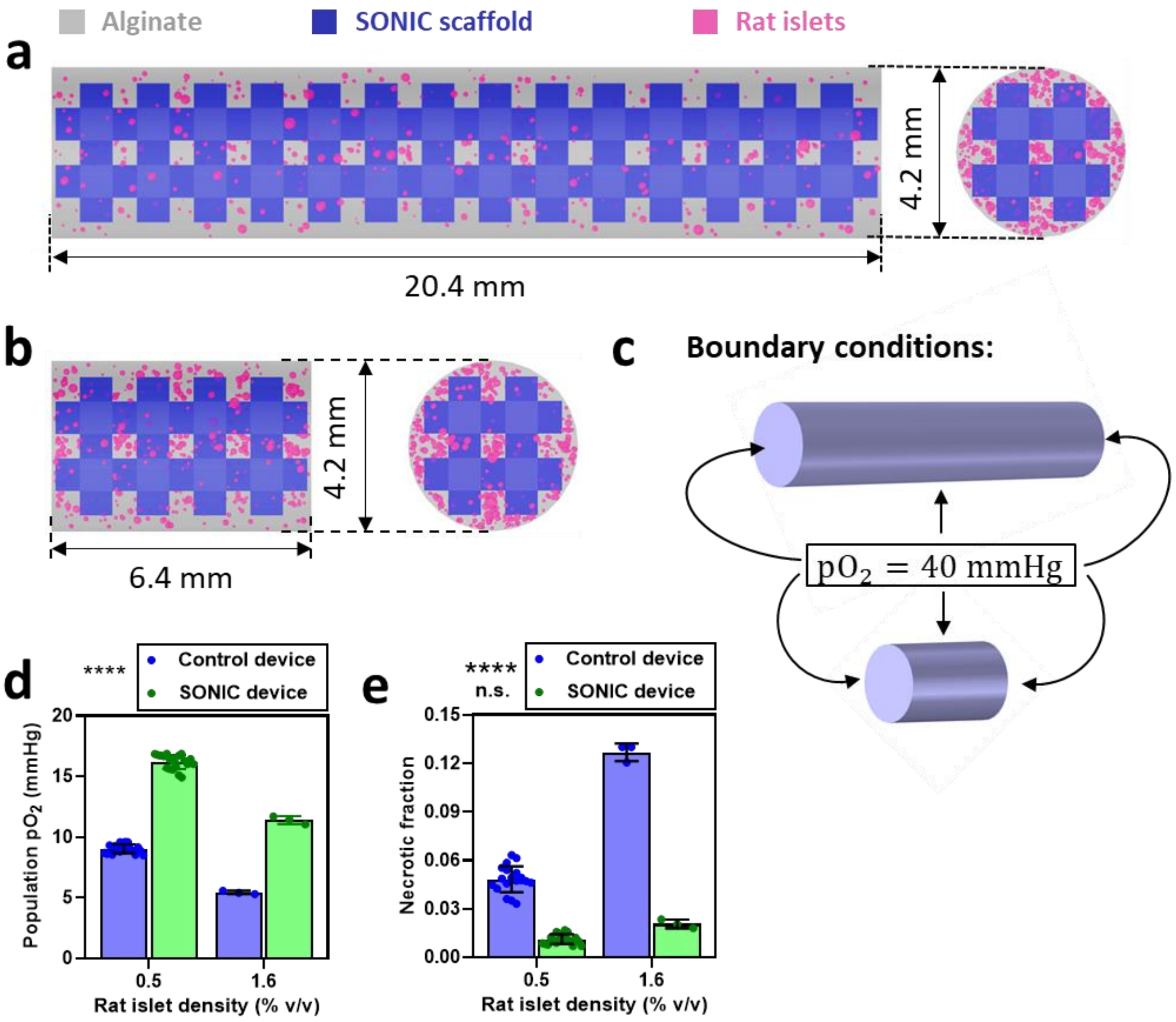

Supplementary Fig. 11 | Simulation-predicted performance of a SONIC device with 500 IEQ rat islets in cylindrical devices at different cell densities. **a**, **b** Annotated schematics of the 20.4 mm length SONIC device (**a**) and the 6.4 mm length SONIC device (**b**). **c** Boundary conditions: a constant pO_2 of 40 mmHg was applied to all surfaces of both devices. **d**, **e** Simulation predictions of the mean islet population pO_2 (**d**) and necrotic fraction of islet tissue (**e**) within empty control devices (blue, n = 20) versus SONIC devices (green, n = 3) at the tested lengths, mean ± SD; d: ****p < 0.0001 (all comparisons); e: n.s. (p = 0.0770) (SONIC device, 20.4 mm versus 6.4 mm), ****p < 0.0001 (all other comparisons). Statistical tests in d and e were analyzed via a two-way ANOVA followed by Sidak's post hoc p-value adjustment for multiple comparisons.

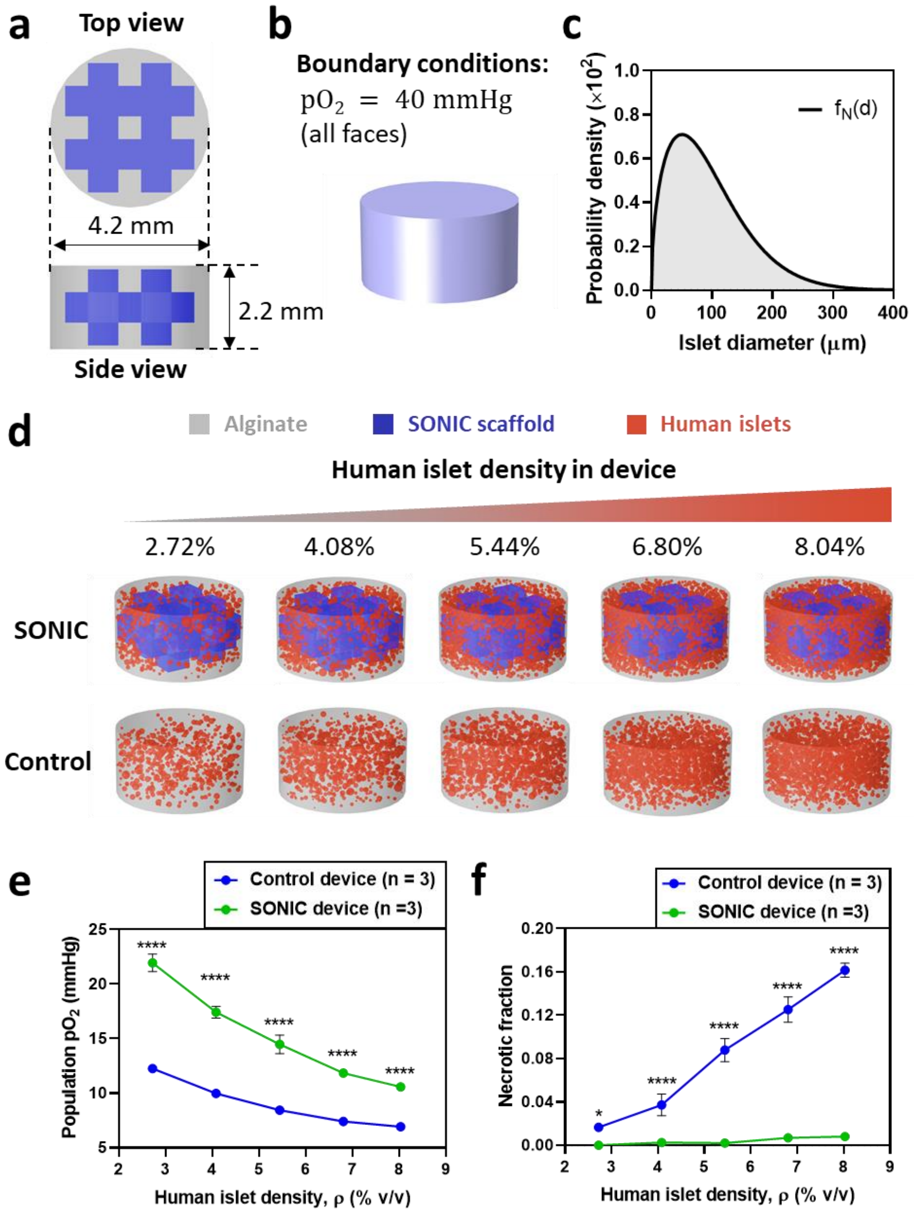

Supplementary Fig. 12 | Simulation-predicted performance of a SONIC device with human islets at variable loading density (volume of islets per volume of device). **a** Annotated schematic depicting the dimensions of the SONIC device containing human islets at variable densities. **b** Boundary conditions: a constant pO_2 of 40 mmHg was implemented on all faces. **c** (Number basis) probability density function (f_N) of the size distribution of human islets. **d** Schematic illustrating the SONIC device and empty control device encapsulating increasing densities of human islets. **e**, **f** Simulation predictions of the mean islet population pO_2 (**e**) and necrotic fraction (**f**) of human islets in empty control devices (blue) versus SONIC devices (green) at the tested densities. Densities were given as volume of islets per volume of device, mean ± SD; e: ****$p < 0.0001$ (control device versus SONIC device at all densities); f: *$p = 0.0294$ (control device versus SONIC device, at density of 2.72%), ****$p < 0.0001$ (control device versus SONIC device, all other densities). Statistical tests in e and f were analyzed via a two-way ANOVA followed by Sidak's post hoc *p*-value adjustment for multiple comparisons.

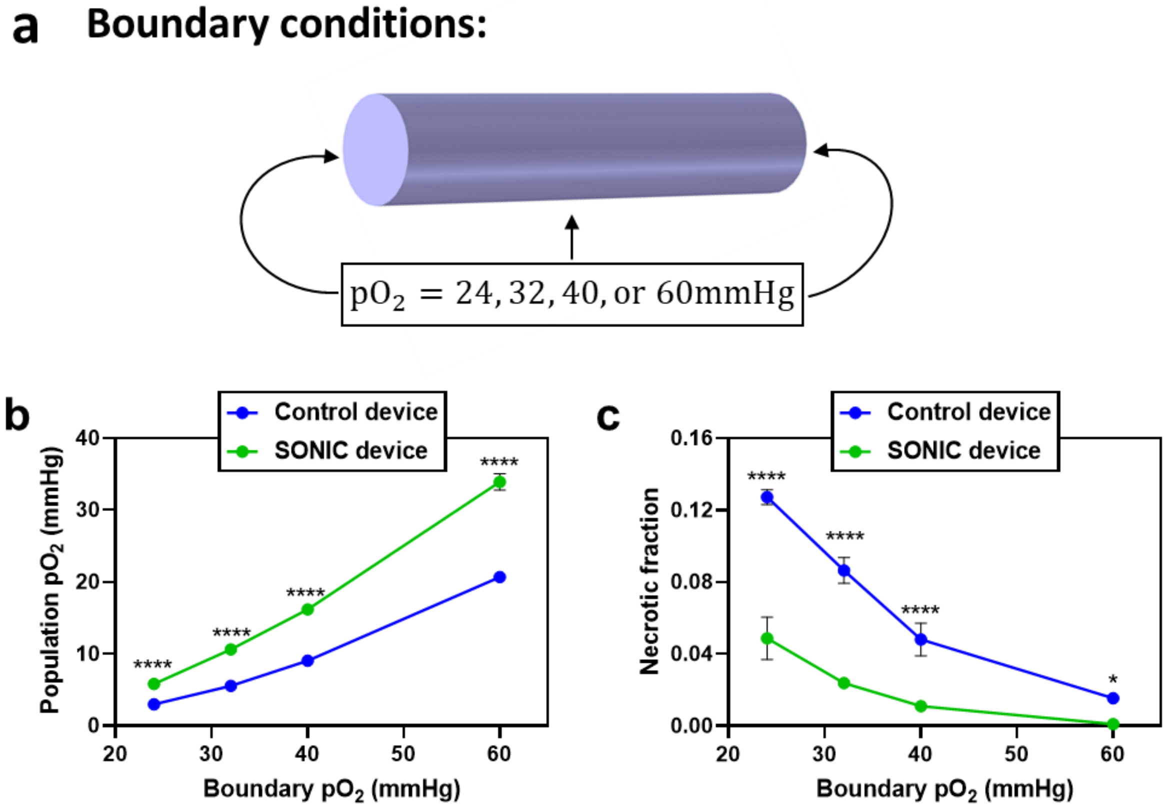

Supplementary Fig. 13 | Effect of variable external pO_2 on simulated rat islet (500 IEQ) oxygenation in the SONIC device (4.2 mm in diameter, 20.4 mm in length) versus the control device. **a** Boundary conditions: a series of pO_2 were implemented on all faces. **b**, **c** Simulation predictions of the mean islet population pO_2 (**b**) and necrotic fraction (**c**) in the control device and SONIC device, (n =3 for boundary pO_2 of 24 mmHg, n =3 for boundary pO_2 of 32 mmHg, n =20 for boundary pO_2 of 40 mmHg, and n =3 for boundary pO_2 of 60 mmHg), mean ± SD; b: $****p < 0.0001$ (all comparisons); c: $*p = 0.0215$ (control device versus SONIC device, 60 mmHg), $****p < 0.0001$ (control device versus SONIC device, all other boundary pO_2 settings). Statistical tests in b and c were analyzed via a two-way ANOVA followed by Sidak's post hoc *p*-value adjustment for multiple comparisons.

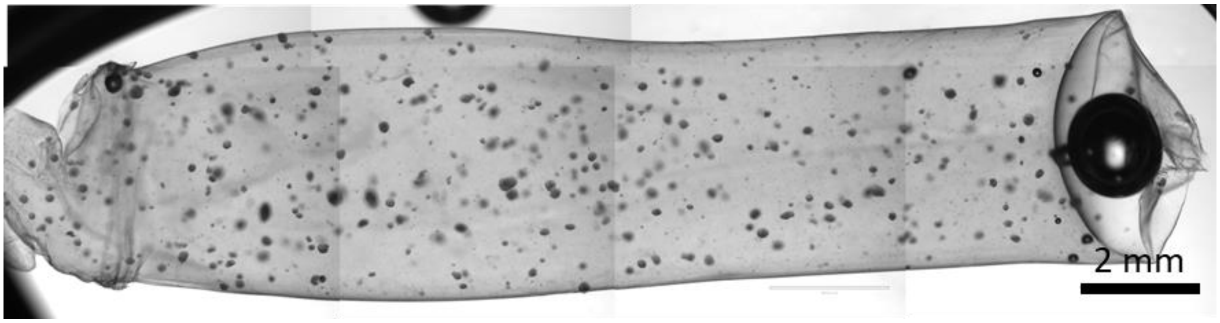

Supplementary Fig. 14 | Microscope image of the rat islets encapsulated control device (~4 mm in diameter). One representative of 5 replicates is shown.

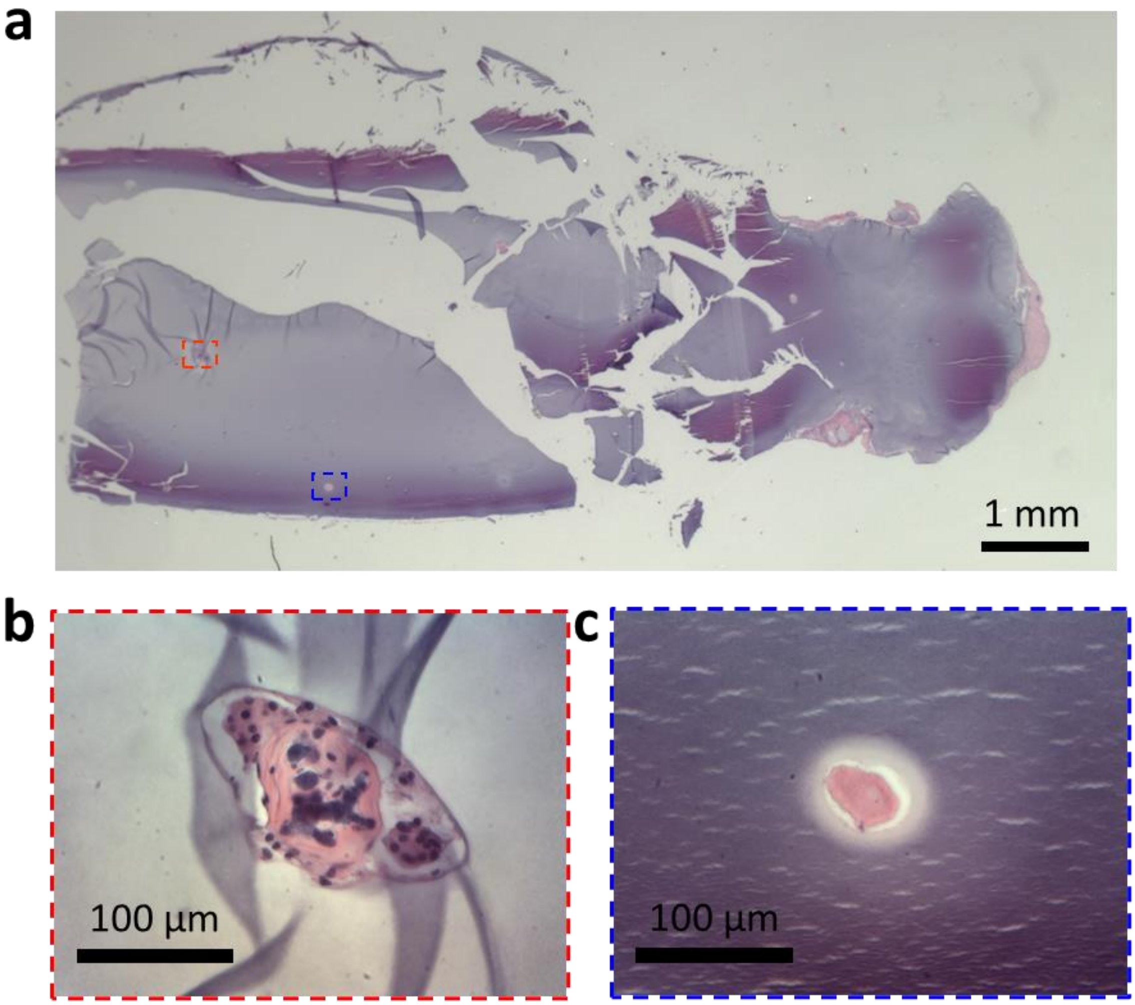

Supplementary Fig. 15 | a–**c** Microscope images of H&E staining of a retrieved control device (**a**) showing necrosed islets with severe karyorrhexis (**b**) or complete loss of nuclei (**c**). One representative of 5 replicates is shown.

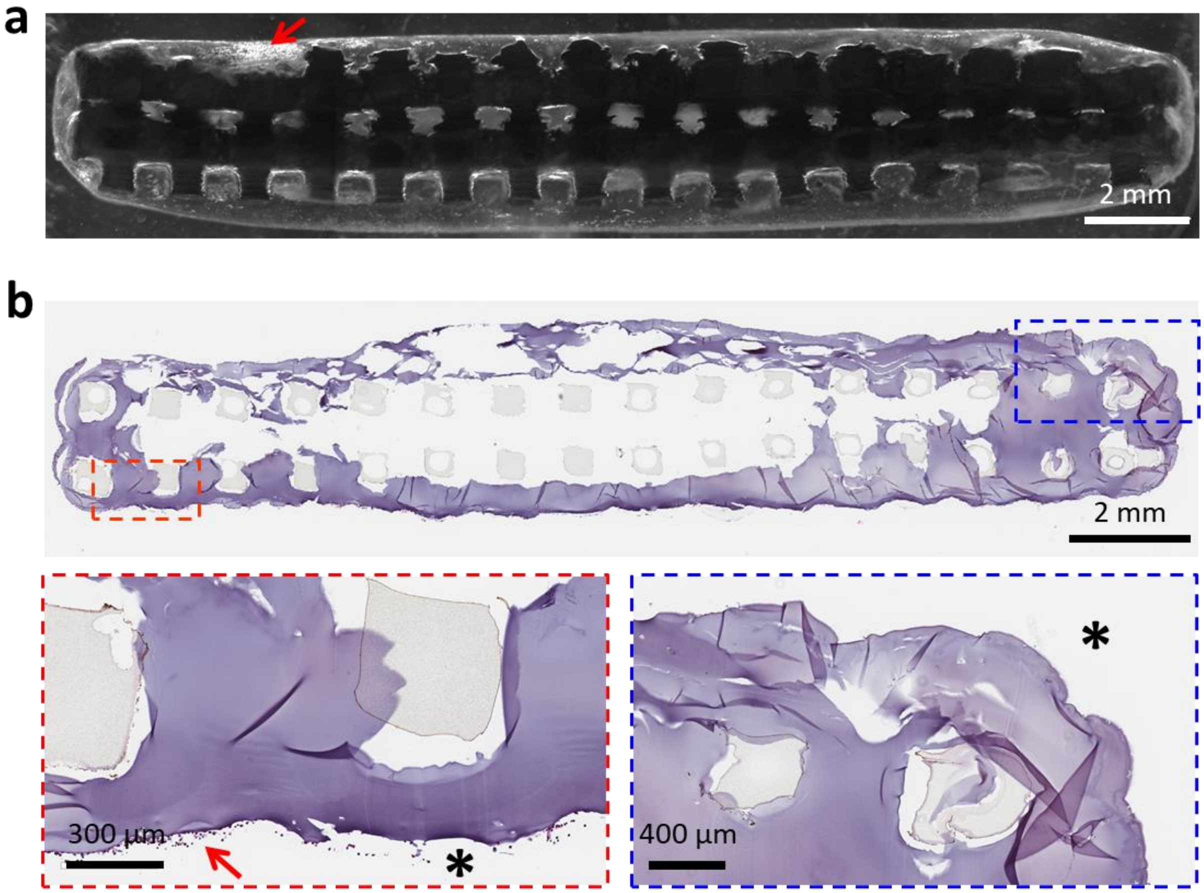

Supplementary Fig. 16 | a, **b** Microscope image (**a**) and H&E staining (**b**) of a retrieved acellular SONIC device after 4-week implantation. The asterisk indicates host side of the device–host interface, and the arrows indicate the minimal cellular overgrowth on the device. One representative of 3 replicates is shown.

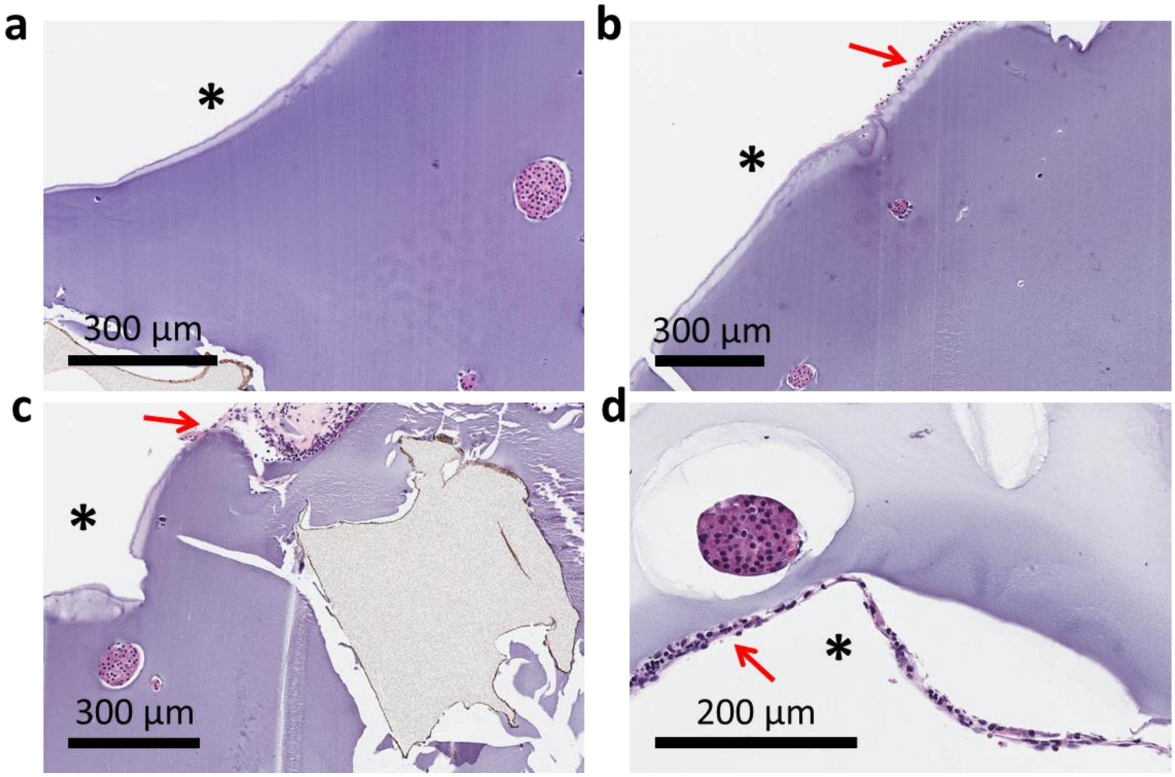

Supplementary Fig. 17 | a–d H&E staining of retrieved SONIC devices after 60-day implantation. The asterisks indicate host side of the device–host interface, and the arrows indicate the mild cellular overgrowth on the device. One representative of 3 replicates is shown.

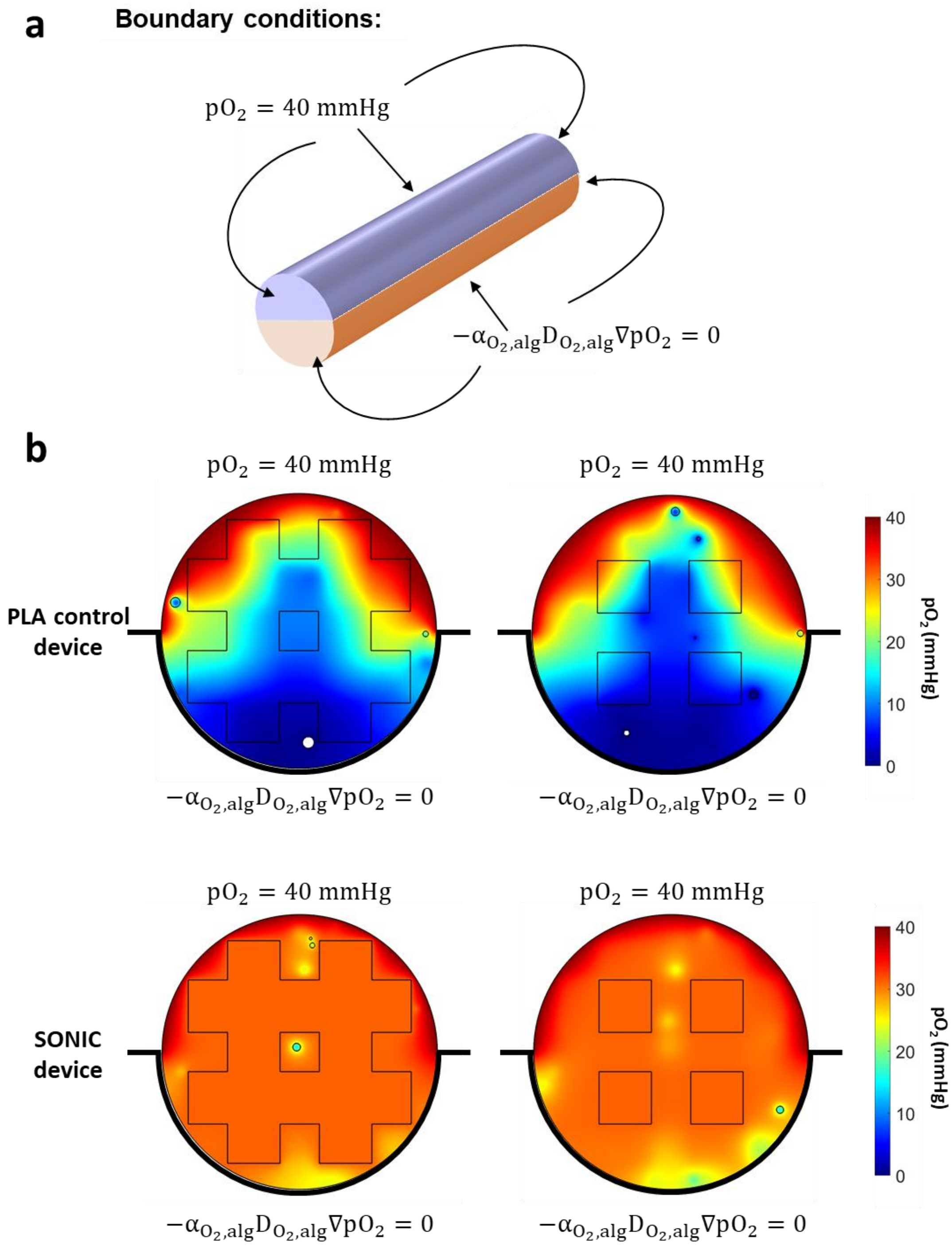

Supplementary Fig. 18 | a Boundary conditions simulating partial fibrotic coverage: it was assumed that one face of the device (4.2 mm in diameter, 20.4 mm in length; 500 IEQ rat islets per device) was constant at 40 mmHg (top) whereas the other was totally blocked by fibrosis (bottom), implemented as a no-flux boundary. **b** Selected transverse surface plots from the partial fibrosis simulation showing severe hypoxic regions and necrotic islets (white regions) near the no-flux interface in the PLA control device, whereas a relatively high and uniform pO_2 profile is observed in SONIC devices, despite the blockage.

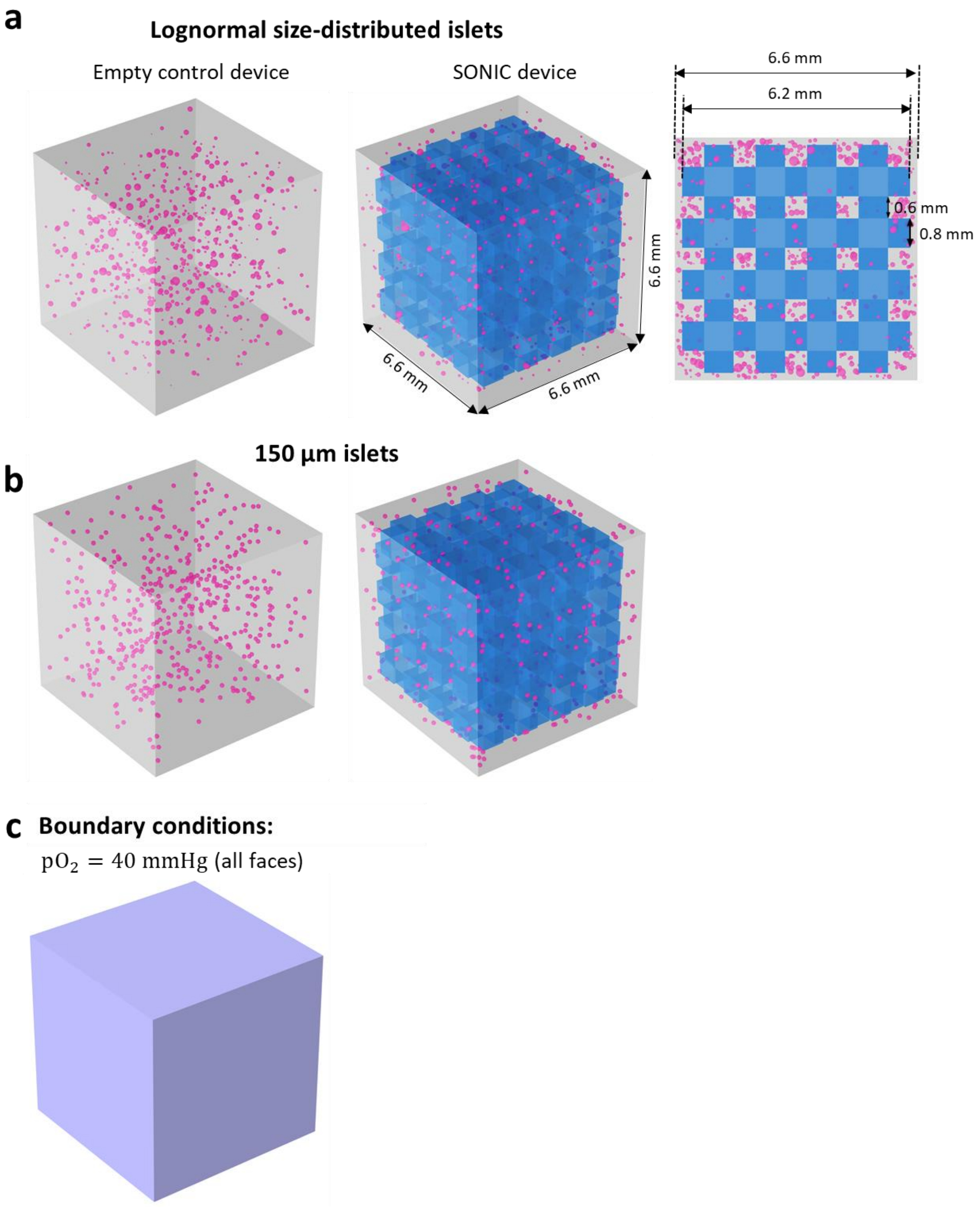

Supplementary Fig. 19 | a Schematic showing the geometry of the thick cubic empty control (left), SONIC device (middle) and annotated dimensions (right) of simulations featuring size-distributed islets (Fig. 6a–d). **b** Schematic showing the geometry of the thick cubic empty control (left) and SONIC device (right) model with uniform (150 µm) islets (Fig. 6e, f). **c** Boundary conditions: in all thick device simulations, all faces were assumed to be a constant pO_2 of 40 mmHg.

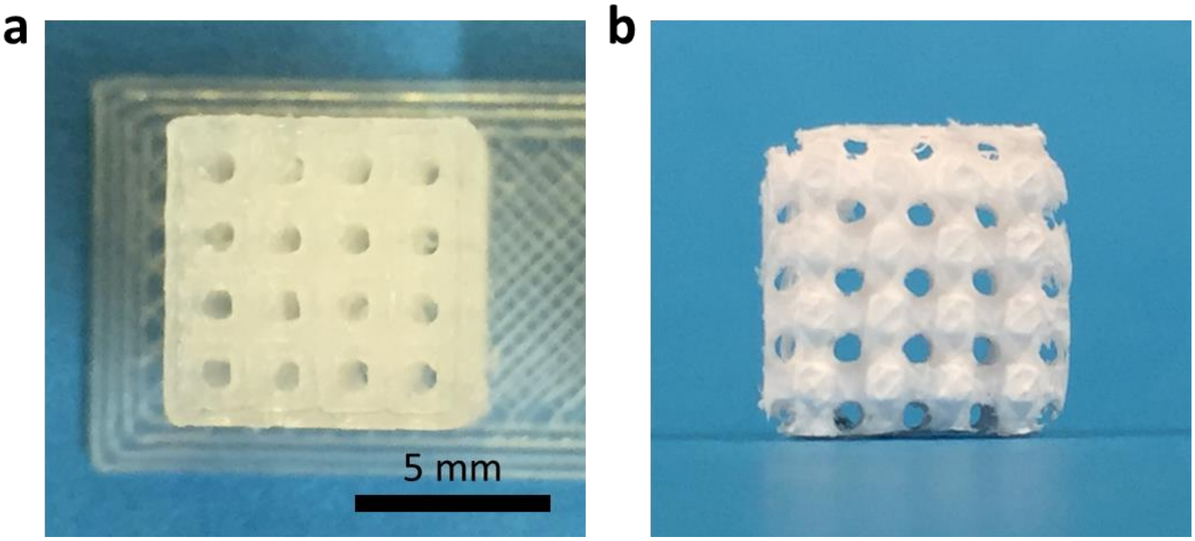

Fig. S20 | a, **b** Digital images of the 3D-printed PLA mold (**a**) for fabrication of the four-layer thick SONIC (**b**).

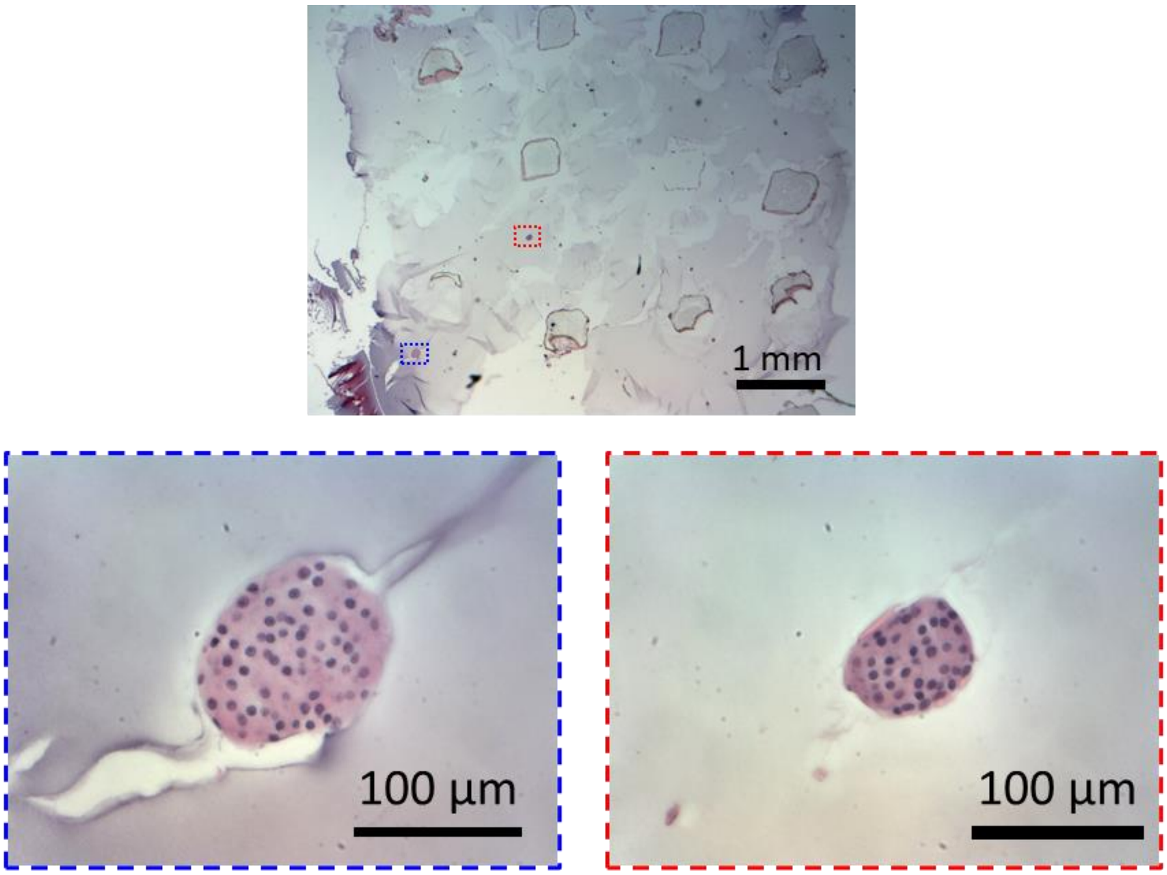

Supplementary Fig. 21 | H&E staining of a retrieved thick cubic SONIC device (with side length of ~6.6 mm) after 4-month implantation. One representative of 5 replicates is shown.

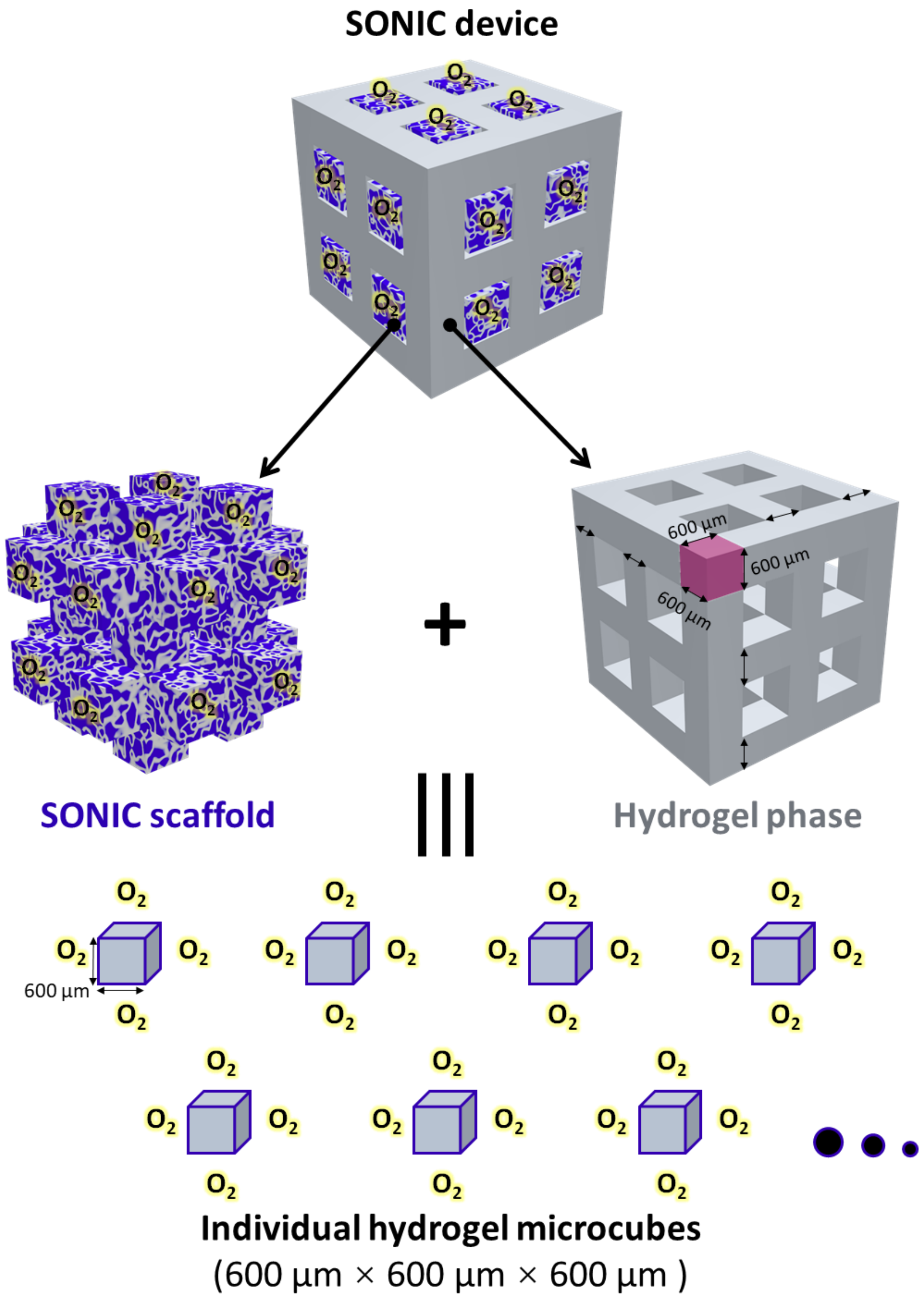

Supplementary Fig. 22 | Schematic of a unit in the SONIC device illustrating the intertwined SONIC scaffold and hydrogel phase, and individually analogic microcubes with surrounding O_2 from the transplantation site.

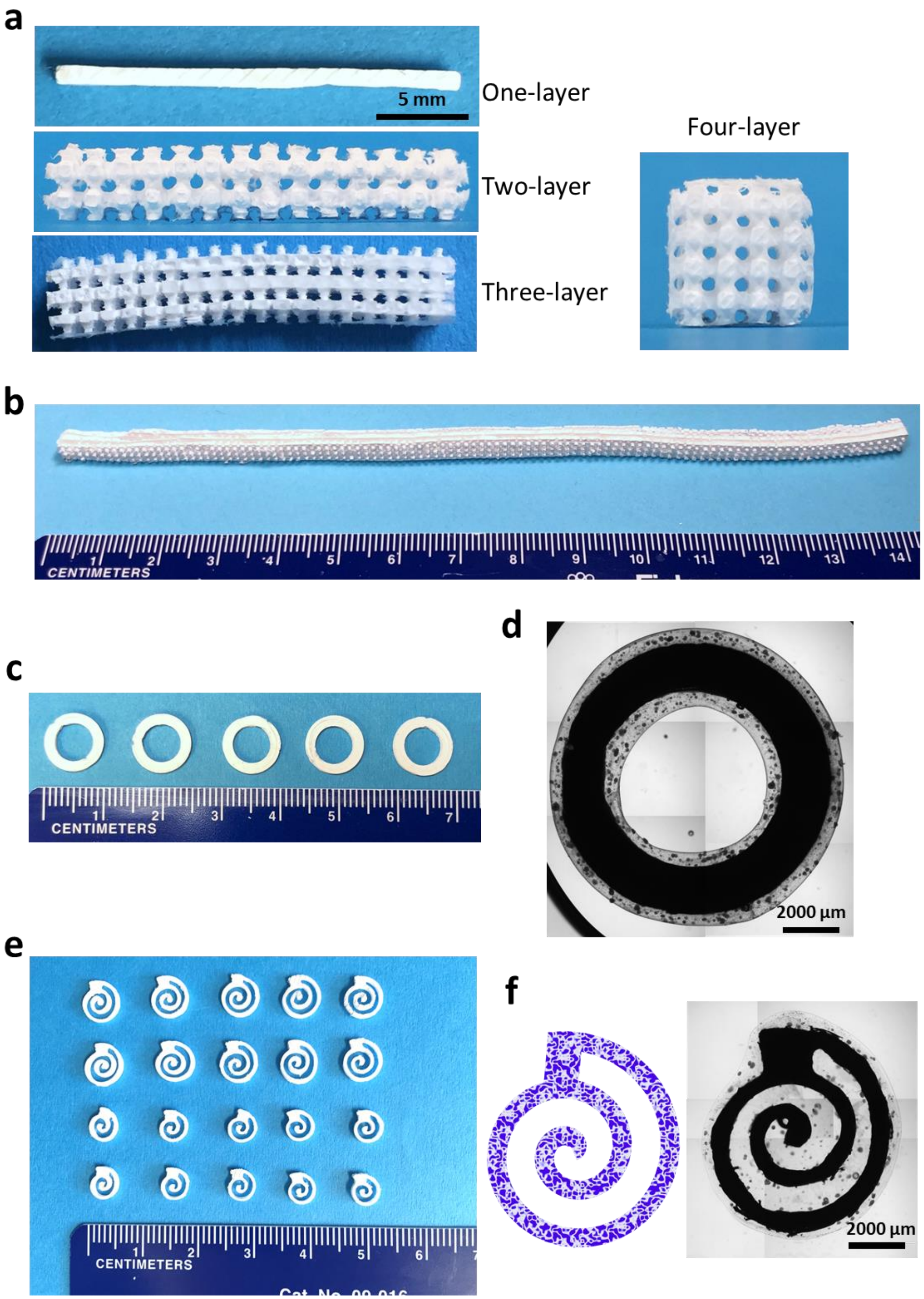

Supplementary Fig. 23 | a Digital images of SONIC scaffolds from one layer to four layers. **b** Digital image of a scaled up three-layer SONIC scaffold with length of ~15 cm. **c** Digital image of a toroidal SONIC scaffold. **d** Microscopy image of a toroidal SONIC device with rat islets. One representative of 3 replicates is shown. **e** Digital image of spiral SONIC scaffolds. **f** Microscopy image of a spiral SONIC device with rat islets. One representative of 3 replicates is shown.

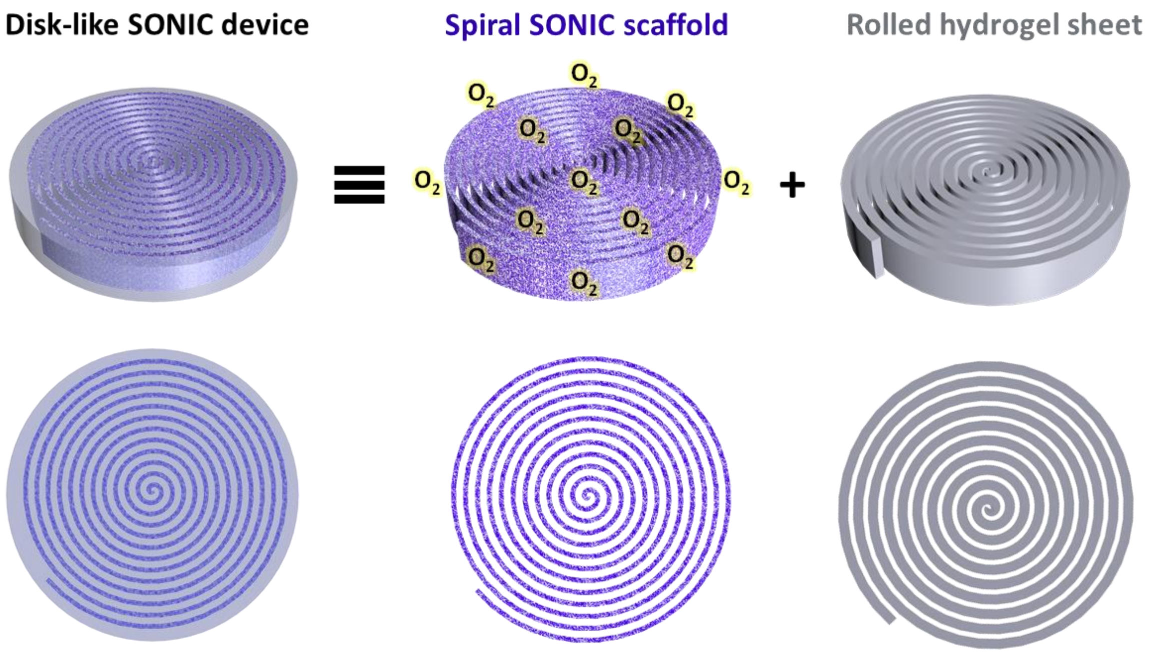

Supplementary Fig. 24 | Schematic of a disk-like SONIC device illustrating the intertwined spiral SONIC scaffold and hydrogel phase (analogous to a rolled hydrogel sheet).

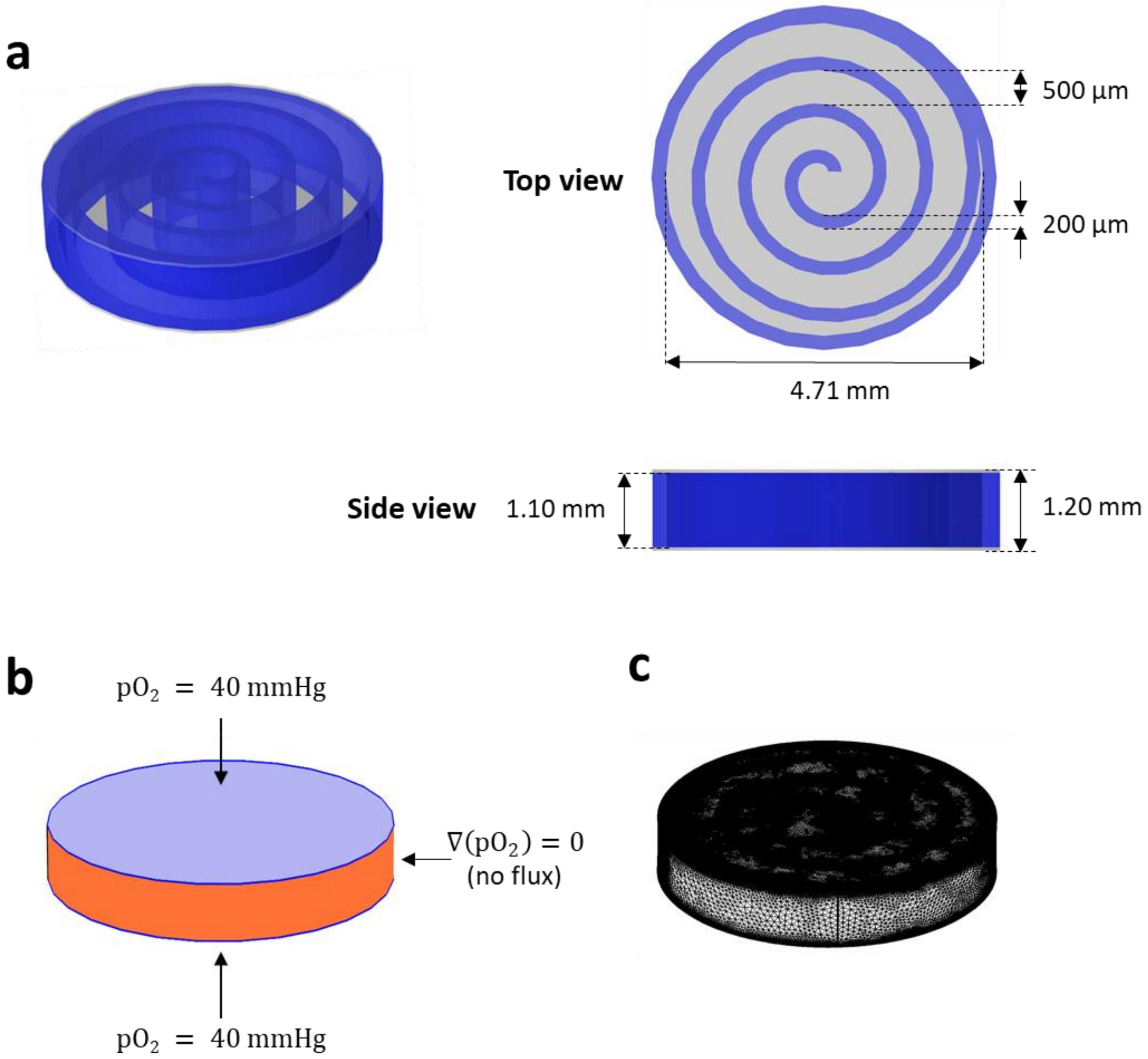

Supplementary Fig. 25 | The scalable spiral SONIC device design. **a** Annotated schematic depicting the dimensions of a planar device featuring a SONIC scaffold in an Archimedean spiral configuration used for simulations. **b** O_2 boundary conditions: a constant external pO_2 of 40 mmHg was applied to the top and bottom faces, and a no-flux condition imposed on the lateral face to mimic negligible edge effects of a device of similar configuration but radially extended. **c** Image of the nonuniform mesh implemented in the simulation.
