# Supplementary material for: A bioinspired scaffold for rapid oxygenation of cell encapsulation systems
Source: Nat Commun. 2021 Oct 6;12:5846. doi: 10.1038/s41467-021-26126-w (PMC8494927; doi:10.1038/s41467-021-26126-w)
Supplement: Supplementary file 3 — Description of Additional Supplementary Files [file 41467_2021_26126_MOESM3_ESM.docx]

Description of Additional Supplementary Files

Title: Supplementary Movie 1

Description: Schematic rotation showing the 3D macrostructure of the ladder-like SONIC scaffold.

Title: Supplementary Movie 2

Description: 3D reconstruction of Nano-CT images of a selected region (24.12 × 35.40 × 5.36 µm) inside the SONIC scaffold showing the bicontinuous microstructure (the diffuse red coloring indicates the air phase in the porous skeleton).

Title: Supplementary Movie 3

Description: A video showing the super-hydrophobicity of the SONIC scaffold.

Title: Supplementary Movie 4

Description: A video showing the simulated real time oxygenation in the system with the control PLA insert and SONIC scaffold and the corresponding average pO2 change in gelatin.
