# Supplementary material for: A bioinspired scaffold for rapid oxygenation of cell encapsulation systems
Source: Nat Commun. 2021 Oct 6;12:5846. doi: 10.1038/s41467-021-26126-w (PMC8494927; doi:10.1038/s41467-021-26126-w)
Supplement: Supplementary file 8 — Reporting Summary [file 41467_2021_26126_MOESM8_ESM.pdf]

Corresponding author(s): Minglin Ma
Last updated by author(s): Sep 1, 2021

Reporting Summary

Nature Research wishes to improve the reproducibility of the work that we publish. This form provides structure for consistency and transparency in reporting. For further information on Nature Research policies, see our Editorial Policies and the Editorial Policy Checklist.

Statistics

For all statistical analyses, confirm that the following items are present in the figure legend, table legend, main text, or Methods section.

n/a	Confirmed	
☐	☒	The exact sample size (n) for each experimental group/condition, given as a discrete number and unit of measurement
☐	☒	A statement on whether measurements were taken from distinct samples or whether the same sample was measured repeatedly
☐	☒	The statistical test(s) used AND whether they are one- or two-sided<br>*Only common tests should be described solely by name; describe more complex techniques in the Methods section.*
☐	☒	A description of all covariates tested
☐	☒	A description of any assumptions or corrections, such as tests of normality and adjustment for multiple comparisons
☐	☒	A full description of the statistical parameters including central tendency (e.g. means) or other basic estimates (e.g. regression coefficient) AND variation (e.g. standard deviation) or associated estimates of uncertainty (e.g. confidence intervals)
☐	☒	For null hypothesis testing, the test statistic (e.g. F, t, r) with confidence intervals, effect sizes, degrees of freedom and P value noted<br>*Give P values as exact values whenever suitable.*
☒	☐	For Bayesian analysis, information on the choice of priors and Markov chain Monte Carlo settings
☒	☐	For hierarchical and complex designs, identification of the appropriate level for tests and full reporting of outcomes
☒	☐	Estimates of effect sizes (e.g. Cohen's d, Pearson's r), indicating how they were calculated

Our web collection on statistics for biologists contains articles on many of the points above.

Software and code

Policy information about availability of computer code

Data collection	Microscopy images were taken by a digital inverted microscope (EVOS FL) using EVOS AMF4300 imaging system.<br>Confocal images were taken by a Zeiss710 using Zeiss Zen 2012 software.<br>H&E staining images were taken by an Aperio Scanscope (CS2) using ISCapture 3.9 software.<br>Stereo microscope images were taken by a stereomicroscope (Olympus SZ61) using ISCapture 3.9 software.
Data analysis	OriginPro 8.5.1 software and GraphPad Prism 8 software were used for data plotting.<br>R 4.1.1 software was used for statistical analyses.<br>COMSOL Multiphysics 5.4 software and MATLAB 2019a software were used for computational modeling.<br>MATLAB 2019a software was used for EPR image processing.<br>ImageJ 1.52p software was used for confocal image processing.<br>Avizo 8.1.1 software was used for Nano-CT image processing.

For manuscripts utilizing custom algorithms or software that are central to the research but not yet described in published literature, software must be made available to editors and reviewers. We strongly encourage code deposition in a community repository (e.g. GitHub). See the Nature Research guidelines for submitting code & software for further information.

Data

Policy information about availability of data

All manuscripts must include a data availability statement. This statement should provide the following information, where applicable:

- Accession codes, unique identifiers, or web links for publicly available datasets
- A list of figures that have associated raw data
- A description of any restrictions on data availability

All data supporting the findings of this study are available within the article and the supplementary information files and from the corresponding author upon reasonable request.

Field-specific reporting

Please select the one below that is the best fit for your research. If you are not sure, read the appropriate sections before making your selection.

☒ Life sciences ☐ Behavioural & social sciences ☐ Ecological, evolutionary & environmental sciences

For a reference copy of the document with all sections, see nature.com/documents/nr-reporting-summary-flat.pdf

Life sciences study design

All studies must disclose on these points even when the disclosure is negative.

Sample size	The chosen sample sizes in this study were similar to those generally employed and accepted in this field (see references: Song et al., Nature Communications, 2019, 10 (4602), 1–12; Liu et al., Nature Communications, 2019, 10 (5262), 1–14; and Wang et al., Sci. Adv. 2021, 7, eabd5835.)<br>All sample sizes are indicated in the figure legends, and the sample sizes were sufficient to conduct reasonable statistical analyses where applicable.
Data exclusions	No data was excluded.
Replication	All experiments were performed at least twice, but more often more than three times.<br>Details about the replication of experiments are indicated in the figure legends where applicable.
Randomization	For in vitro studies, the same cells/alginate suspension was used for the control group and SONIC group, then some samples from each group were randomly chosen for following staining and imaging.<br>For in vivo studies, mice were randomly allocated by body weight and level of diabetic state (blood glucose level after diabetes induction) to the different experimental groups.<br>For ex vivo studies, samples were randomly chosen for following staining and imaging.
Blinding	No formal blinding was used. For the in vitro and ex vivo characterizations, the SONIC devices can be easily identified by the visual observation of the inner incorporated scaffold. For the in vivo characterizations, it's easy to recognize the control device groups by the wet cages from frequent urination of uncorrected diabetic mice. However, the blood glucose monitoring was performed by different individuals and the GSIS ELISA measurement was performed in a blinding way.

Reporting for specific materials, systems and methods

We require information from authors about some types of materials, experimental systems and methods used in many studies. Here, indicate whether each material, system or method listed is relevant to your study. If you are not sure if a list item applies to your research, read the appropriate section before selecting a response.

Materials & experimental systems

n/a	Involved in the study	
☐	☒	Antibodies
☐	☒	Eukaryotic cell lines
☒	☐	Palaeontology and archaeology
☐	☒	Animals and other organisms
☒	☐	Human research participants
☒	☐	Clinical data
☒	☐	Dual use research of concern

Methods

n/a	Involved in the study	
☒	☐	ChIP-seq
☒	☐	Flow cytometry
☒	☐	MRI-based neuroimaging

Antibodies

Antibodies used	Rabbit anti-rat insulin (Abcam, ab63820, polyclonal, 1:200)

Antibodies used	mouse anti-rat glucagon (Abcam, ab10988, clone K79bB10, 1:200)<br>Alexa Fluor 594-conjugated goat anti-rabbit IgG (Thermofisher, A11037, 1:400)<br>Alexa Fluor 488-conjgated donkey anti-mouse IgG (Thermofisher, A21202, 1:400)
Validation	All antibodies have been validated for target specificity and applications by the manufacturers and published literature.<br>Rabbit anti-rat insulin<br>(https://www.abcam.com/insulin-antibody-ab63820.html and and Wang et al., Sci. Adv. 2021; 7 : eabd5835);<br>mouse anti-rat glucagon<br>(https://www.abcam.com/glucagon-antibody-k79bb10-ab10988.html and Wang et al., Sci. Adv. 2021; 7 : eabd5835);<br>Alexa Fluor 594-conjugated goat anti-rabbit IgG<br>(https://www.thermofisher.com/antibody/product/Goat-anti-Rabbit-IgG-H-L-Highly-Cross-Adsorbed-Secondary-Antibody-Polyclonal/A-11037 and Wang et al., Sci. Adv. 2021; 7 : eabd5835);<br>Alexa Fluor 488-conjgated donkey anti-mouse IgG (https://www.thermofisher.com/antibody/product/Donkey-anti-Mouse-IgG-H-L-Highly-Cross-Adsorbed-Secondary-Antibody-Polyclonal/A-21202 and Wang et al., Sci. Adv. 2021; 7 : eabd5835).

Eukaryotic cell lines

Policy information about cell lines

Cell line source(s)	INS-1 cells were purchased from Sigma-Aldrich.
Authentication	The vendor had performed cell authentication.
Mycoplasma contamination	Cells tested negative for mycoplasma contamination.
Commonly misidentified lines<br>(See ICLAC register)	No commonly misidentified lines were used in this study.

Animals and other organisms

Policy information about studies involving animals; ARRIVE guidelines recommended for reporting animal research

Laboratory animals	8-week-old male C57BL/6J mice (Stock No: 000664) were purchased from the Jackson Laboratory (Bar Harbor, ME).<br>The mice were maintained at a temperature of 70-72°F with 30-70% humidity under a 14-hour light/10-hour dark cycle.<br>8-week-old male Sprague-Dawley rats (Strain Code 400, weight ~300 g) were purchased from Charles River Laboratories (Wilmington, MA).<br>The rat were maintained at a temperature of 70-72°F with 30-70% humidity under a 12-hour dark/12-hour light cycle.<br>The mealworm beetles (Tenebrio molitor) were purchased from PetSmart (Ithaca, NY).
Wild animals	Studies did not involve wild animals
Field-collected samples	No studies involved samples collected from the field.
Ethics oversight	The Cornell Institutional Animal Care and Use Committee approved all animal procedures.

Note that full information on the approval of the study protocol must also be provided in the manuscript.
